# Inhibition of quorum sensing-controlled virulence factor production in *Pseudomonas aeruginosa* by *Quercus infectoria* gall extracts

**Published:** 2017-02

**Authors:** Samaneh Mohabi, Davood Kalantar-Neyestanaki, Shahla Mansouri

**Affiliations:** 1 Department of Microbiology and Virology, School of Medicine, Kerman University of Medical Sciences, Kerman, Iran; 2 Research Center for Infectious and Tropical Diseases, Kerman University of Medical Sciences, Kerman, Iran

**Keywords:** *Pseudomonas aeruginosa*, *Quercus infectoria*, Virulence factor, Quorum sensing, *LasR* gene, *Chromobacterium violaceum*

## Abstract

**Background and Objectives:**

This study was designed to evaluate the activity of *Quercus infectoria* galls extract (QIFGE) on virulence factor production and inhibition of quorum sensing (QS) in *Pseudomonas aeruginosa.*

**Materials and Methods:**

Minimum inhibitory concentration (MIC) of QIFGE against 5 strains of *P. aeruginosa* was determined. The extract at sub-MIC was used to determine biofilm formation, level of protease LasA, LasB, swarming and twitching motility and QS using *Chromobacterium violaceum* CV026 as a biosensor. Effect of the extract on expression levels of *lasR* gene was determined by real time PCR.

**Results:**

QIFGE inhibited the QS and all other tested virulence factors compared with the control grown in the absence of the extract (P=0.001). Real time PCR showed 2 to 8-fold reduction in *lasR* gene expression in presence of the extracts compared with the control. QIFGE significantly inhibited the virulence factor production, had inhibitory effect on QS, and resulted in the lower expression of *lasR* gene.

**Conclusion:**

QIFGE showed novel inhibitory effect against QS related virulence factor production, which was unrelated to antimicrobial effect. The extract can down regulate the production of virulence factor and should be evaluated as a candidate for alternative treatment of pseudomonad infections in future.

## INTRODUCTION

*Pseudomonas aeruginosa* is ubiquitous in nature and can be isolated from various environments. As an opportunistic pathogen it is capable of producing different types of infection, especially in patients with compromised host defense. Apart from nosocomial infections, this bacterium can cause a series of infections such as chronic lung infection in patients with cystic fibrosis, bacteremia in severe burn victims, and acute ulcerative keratitis in users of extended-wear soft contact lenses (1, 3). *P. aeruginosa* strains are able to build up resistance to disinfectants and have the intrinsic or acquired resistance to antimicrobial agents, and all strains are prone to becoming multiple drug resistant (MDR) (1, 4). In *P. aeruginosa* and many other bacteria, cell to cell communication or quorum-sensing (QS) is important in the bacterial pathogenicity (5). N-acyl homoserine lactone (AHL) are the autoinducers in this system, and because of their small size and their lipophilic characters freely diffuses across the membrane (5, 6). As the population density increases, intracellular AHL binds to the corresponding receptor at a sufficient concentration within the cytoplasm to induce differential gene expression (5, 6). To date, three types of QS systems including *las, rhl* and *pqs* have been identified in *P. aeruginosa*, production of *las* and *rhl* system are related to AHL signaling (6). In *P. aeruginosa*, the *lasR* gene is required for transcription of two proteases which are associated with bacterial virulence, namely, elastase (LasB) and protease (LasA). It has been suggested that *lasR* gene plays a global role in the pathogenicity of *P. aeruginosa* (8). Drug resistance, especially emergence of MDR strains of *P. aeruginosa* is an increasing problem worldwide, causes problems in the treatment of infections, and is usually associated with significant morbidity and mortality (9). Low outer membrane permeability, expression of several efflux pumps and production of different extracellular enzymes are the main cause of resistance in this bacterium (5, 9).

Medicinal plants have evolved numerous special chemical strategies to inhibit and suppress the bacterial pathogens, including the production of bactericidal and anti-infective compounds, leading to their contribution to biology and medicine (10, 11). Recently researchers have paid special attention to the use of medicinal plants mostly by inhibiting or reducing the pathogenicity of bacteria rather than killing the agents (12–15). Plant extracts with quorum quenching activities could be considered as a promising and novel target for development of anti-pathogenic drugs, especially in the struggle with infections caused by resistance strains. A number of medicinal plants have anti-QS potential, and their effect on virulence factors production, such as biofilm formation, expression of QS gene and auto inducer production has been reported. (11, 13, 14). *Quercus infectoria* Oliver (Family: Fagaceae), also known as gall oak, is a small shrub found in Iran, Greece and Asia Minor (15). The extract of insect gall oak is used as a traditional medicine, and is proposed to have many therapeutic properties such as antiviral, antibacterial, larvicidal and antibiofilm activities (15, 16). In view of diverse medicinal applications of this plant, and the observation that phytochemicals from plants can inhibit QS activities, this investigation was designed to evaluate the inhibitory effect of QIFGE on virulence factor production and the expression of *lasR* gene, which is an important factor in QS activities in *P. aeruginosa.*

## MATERIALS AND METHODS

### Plant material.

The galls of *Quercus infectoria* were obtained from commercial source. The identity of the plant was confirmed with the help of scientists in the Department of Botany of Kerman University. A voucher specimen was deposited in the Herbarium of Kerman Faculty of Pharmacy, Kerman, Iran. *Quercus infectoria* is kept at the Kerman University of Medical Science. The plant galls were washed, dried and ground to a fine powder.

### Extraction procedure.

The crude plant extracts were obtained by extracting dried material (35 gr) with 300 ml absolute methanol (Merck, Germany) for 24 hours. The extracts were then filtered through a Buchner funnel, and the solvent removed on a rotary evaporator with a reduced pressure at 60–65°C. Finally the extract was dried at 50° C and kept at −4°C in desiccators for further use.

### Bacterial strains, growth conditions and culture.

Five strain of *P. aeruginosa* were tested in this study: *P. aeruginosa* ATCC 27853, PAO 1 wild, PAO1 (MH873), strain MS.PS.50/35, and PDO 300 (mucA2e, a hyper alginate producer), for convenience these strains are referred as strain 1 to 5 respectively. The bacterial strains were a kind gift from Prof. Niels Høiby (Copenhagen Denmark). A violacein-negative mini-Tn5 mutant of *Chromobacterium violaceum* ATCC 31532(CV026) unable to produce pigment but the pigment production can be restored by incubation with an external source of N-acylhomoserine lactones (AHLs) was used to detect the anti QS activities of the plant extracts (17). All bacterial strains used in this study were grown on LB agar at 35°C, except the *C. violaceum* CV026 which was incubated at 28°C (17).

### Minimum inhibitory concentration of the QIFGE against *P. aeruginosa strains.*

Agar dilution method was performed according to CLSI guidelines to find the MIC of the extract against 5 strains of *P. aeruginosa* (18). The concentration range of the extract was 50–400 μg ml^−1^, and the MIC was found to be 200 μg ml^−1^ for all bacterial strains, one dilution below the MIC (100 μg ml^−1^) was regarded as the sub-MIC concentration, and was used for evaluation of virulence factor production in this study.

### LasA staphylolytic assay.

LasA staphylolytic activity was performed as described by Kong et al. (5). Briefly, overnight culture of *P. aeruginosa* strains grown on LB medium with or without QIFGE at a final concentration of 100μgml^−1^ (35°C in a shaker incubator, 120 rpm), were centrifuged. The cell pellet was removed; and the protein content of the supernatant was determined by the Bradford assay (19). An overnight culture of *S. aureus* (ATCC 25923) was boiled, the boiled cell pellet was suspended in 10mM Na_2_PO_4_ (pH=4.5) to an optical density of about 0.8 at wavelength 660nm. *LasA* protease activity was determined by adding an aliquot of 100μgml^−1^ of *P. aeruginosa* supernatant to 900 μl of *S. aureus* suspension, and the OD 660 was determined after 60 min (5).

### LasB elastolytic assay.

The culture supernatant was prepared as mentioned for LasA. An aliquot of *P. aeruginosa* culture supernatant (100μl) was added to elastin-Congo red buffer (900 μl, ECR, Sigma, St. Louis). The suspension was incubated with shaking for 3 hours. The insoluble ECR was removed by centrifugation. The absorption of the supernatant was measured at 495 nm (5). Both LasA and LasB activities were expressed as the change in the OD per hour per μg of protein. Negative control of the assay was non cultivated LB medium. All the experiments were repeated 3 times.

### Swarming motility assay.

The efficacy of QIFGE on bacterial motility was tested using modified M9 medium (M8 medium) supplemented with 0.2% glucose and 2 mM MgSO_4_, the medium also contained 0.5% agar with 0.05% glycine as the sole source of nitrogen (20). For the test, the medium was supplemented with 100 μgml^−1^ final concentration of QIFGE. The plates were briefly dried in air and the freshly grown colony of each bacterial strain from LB agar was transferred to the plates by a sterile tooth pick. The plates were incubated at 37°C for 24 hours and the zones of swarm were measured (21). For twitching motility swarm plates were prepared as indicated except that a thin layer of 1℅ LB agar was prepared (approximately 3mm) according to Déziel et al. 2001 (22), the test plate was also supplemented with 100μgml^−1^ final concentration of QIFGE. A colony of bacteria grown up to stationary phase of growth on LB agar was stab inoculated into the bottom of the plate with a sterile toothpick. The plates were incubated for 24 to 48 hours at 30°C, and the hazy zone of growth at the interface between the agar and the polystyrene surface of the plate was measured.

### Static culture biofilm assay.

Biofilm formation was determined according to O,Toole and Kolter.1998 (23). The bacterial strains were grown in LB broth overnight, and the OD of the medium was adjusted (OD at 600nm:1). Crude extract of QIFGE at a final concentration of 100 μgml^−1^ was added to the culture medium, and aliquot of 100 μl was added to the wells of 96-well polystyrene microtiter plate (Falcon ,USA). The plates were incubated at 30°C, after 24 hours incubation the plates were gently washed with water (three times) to remove non adherent cells. Attaching cells were stained with 0.1% crystal violet in water. Biofilm formation in presence or absence of the extract was visualized at OD 595 nm after addition of 200μl of 95% ethanol.

### Assessment of QS inhibition by *C. violaceum* CV026.

The assay was performed on LB agar containing 20μgml^−1^ kanamycin, with or without 100μgml^−1^ of the extract. Each plate was streaked with a strain of *P. aeruginosa* parallel with *C. violaceum* CV026 (17). Anti QS activities were detected when the *C. violaceum* CV026 was not able to produce pigment when grown in the presence of *P. aeruginosa* strains. The plate without the extracts was used as the negative control.

### RNA extraction and quantitative reverse transcription PCR (quantitative RT-PCR).

The quantitative RT-PCR was used to determine the transcription level of *lasR* gene. Bacterial strains were grown in LB broth with or without QIFGE (100μgml^−1^) at 37°C in a shaker incubator (180 rpm) to the late log phase, equal to 0.8–1 absorbance at 600 nm. The cell pellets were collected by centrifugation. Total RNA was extracted with a Hybrid-R™ RNA extraction kit (Thermo Scientific™, GeneAll, General Biotechnology Co., LTD), according to the manufacturer’s recommendations, RNase-Free DNase I (Thermo Scientific™) was used for elimination of DNA contamination. Concentrations and quality of RNA were determined using absorbance of the sample at 250nm (NanoDrop 2000; Thermo Scientific). Reverse transcription (cDNA synthesize) was performed by the Thermo Scientific kit according to the manufacturer’s recommendations. The transcription level of the *lasR* gene was determined by relative quantitative RT-PCR as described using the standard curve method by Real Q Plus 2× Master Mix Green Kit (Ampliqon, Co) in a Thermal Cycler (Bio-RAD Company). Expression of the *rpoD* gene was assessed in parallel to normalize the transcriptional levels of target genes. The transcriptional level of *lasR* was compared between different bacterial strains in presence or absence of the extract. The quantitative RT-PCR of *rpoD* and *lasR* was performed using the following primers: *lasR* F-5′-AAG GAA GTG TTC AAG TGG TG-3′, *lasR* R-5′-CAG TTG CAG ATA ACC GA-3′ (24) and *rpoD*-F-5′-GGGCGAAGAAGGAAATGGTC-3′ and *rpoD*-R-5′-CAGGTGGCGTAGGTGGAGAA-3′ (25). Fold-changes in gene expression were calculated according to the 2^−ΔΔCT^ method (26).

### Statistical analysis.

Statistical analysis was performed using SPSS (version.22.0) statistics software. A difference was considered statistically significant at a P-value of <0.05.

## RESULTS

### Antimicrobial activity of the methanol extract of *Quercus infectoria.*

QIFGE at a concentration of 200 μgml^−1^ inhibited the growth of all bacterial strains tested. At concentration of 100μgml^−1^ the extract had a marginal effect on the colony size and was not inhibitory to the growth. This sub-MIC concentration was used for evaluation of virulence factor production and *lasR* gene expression.

### LasA staphylolytic and LasB elastolytic activities in the presence or absence of QIFGE.

QIFGE at sub-MIC concentration decreased the level of LasA activity ranging from 48% for strain 1 to 65% for strain 4. The level of Las A difference between the test (grown in the presence of the extract) and control (grown in the absence of the extract) was significant (P=0.003), Table1. The level of LasB was reduced from 37% to 65% in presence of QIFGE, which was statistically significant in comparison with the control grown in the absence of the extract (P= 0.001). The difference in the activity of LasB between different strains of *P. aeruginosa* was also significant (P=0.001) (Table1).

### Biofilm formation in the presence or absence of sub-MIC concentration of QIFGE.

A significant decrease in biofilm formation by different bacterial strains was detected in the presence of the sub-MIC concentration of QIFGE, but the activity was not significantly different between bacterial strains tested (Table 1).

**Table 1.** Inhibitory activity of *Quercus infectoria* methanol extract at sub-MIC concentration (100 μgml^−1^) on the level of LasA, LasB and biofilm formation by different strains of *P. aeruginosa*

* Strains	**LasA (Mean±SD)			**LasB (Mean±SD)			***Biofilm (Mean±SD)		

	Test	Control	% Inhibition	Test	Control	% inhibition	Test	Control	% inhibition
**1**	0.19±0.004	0.41±0.009	54	0.3±0.019	0.5±0.011	37	0.70±0.034	1.36±0.47	48
**2**	0.20±0.008	0.39±0.003	48	0.23±0.005	0.57±0.005	59	0.76±0.005	1.68±0.018	54
**3**	0.19±0.003	0.37±0.007	48	0.27±0.019	0.51±0.009	46	0.75±0.009	1.44±0.397	47
**4**	0.20±0.002	0.58±0.005	65	0.19±0.007	0.55±0.011	65	0.76±0.044	1.54±0.038	50
**5**	0.18±0.002	0.4±0.004	53	0.23±0.012	0.47±0.015	50	0.73±0.024	1.67±0.019	56
**P value**	0.003		0.1	0.0006		0.001	0.006		0.6

* Strains of *P. aeruginosa*: 1-ATCC 27853: 2-PAO.wild; 3-PAO1(MH873) ; 4-MS.PS.50/35;5-;PDO300. Test and control: bacterial grown in presence or absence of 100μg ml^−1^ of QIFGE respectively. All experiments were run 3 times.

** LasA and LasB activities were expressed as the mean ± SD of the change in OD_600_ per hour per μg of protein and OD_495_ per μg of protein respectively.

*** Biofilm formation is presented as mean ± SD of OD_595_ of the adhered bacterial cell after staining with crystal violet.

### Swarming and Twitching motility in presence or absence of QIFGE.

**Fig. 1.**
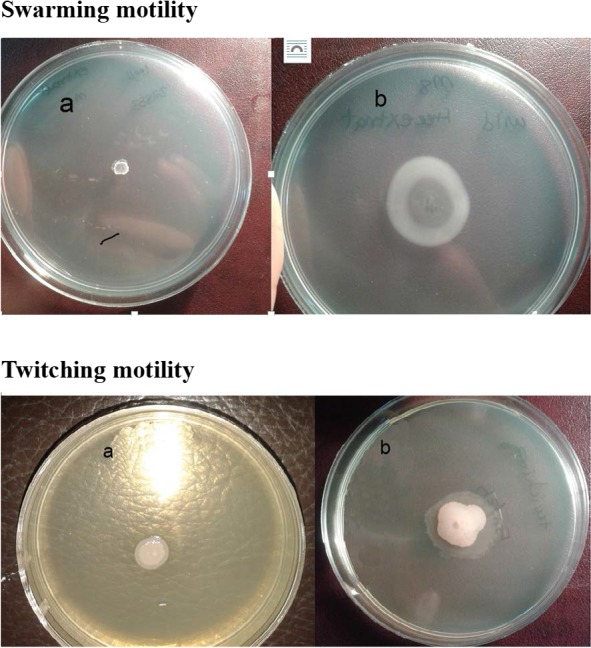
Effect of QIFGE at sub-MIC concentration (100μgml^−1^) on swarming and twitching motility of *P. aeruginosa* in the presence (a) or absence of sub-MIC concentration of QIFGE (100μgml^−1^)

**Fig 2.**
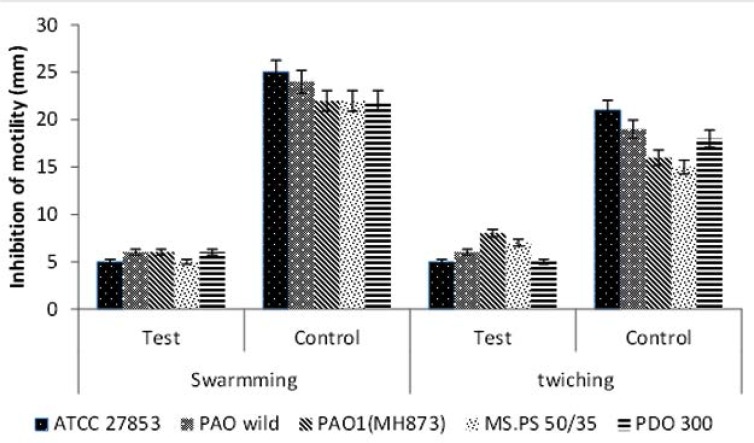
Effect of QIFGE at Sub-MIC concentration (100μgml^−1^) on swarming and twitching motility of *P. aeruginosa.*

The extract at sub-MIC concentration reduced the swarming motility of all the bacterial strains very efficiently and the difference between the test and the control was significant (*P*= 0.0001). The extract also reduced the twitching motility, from 50% to 76%. Effect of QIFGE on twitching motility for different strains of *P. aeruginosa* was found to be statistically significant (Figs. 1 and 2; *P*=0.0001).

For swimming motility M8 medium supplemented with 0.2% glucose and 2 mM MgSO_4_, 0.5% agar and with 0.05% glycine as the sole source of nitrogen was used. For twitching motility the same medium but a thin layer of agar (0.3mm) was used.

Inhibition of motility is expressed as a comparison between swarming or twitching motility in the presense (test) or absence (control) of the QIFGE.

### Detection of anti-QS activities of QIGE using the *C. violaceum* CV026.

**Fig. 3.**
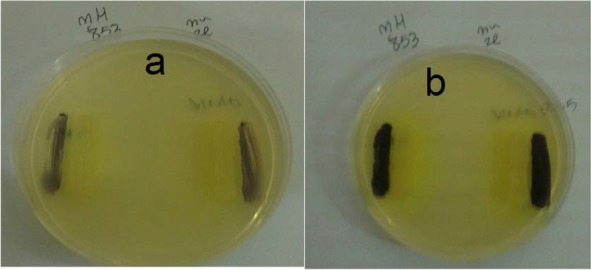
Biosensor plate bioassay showing the induction of violacein in *C. violaceum* CV026 biosensor strain grown in adjacent to *P. aeruginosa* in the presence (a) or absence of sub-MIC concentration of QIFGE (100μgml^−1^)

AHL activity and inhibition of AHL synthesis was determined using *C. violaceum* CV026, which is a mutant strain defective in production of violacein in the absence of exogenous AHL. The results show that none of the bacterial strains grown in presence of sub-MIC concentrations of QIFGE were able to assist *C. violaceum* CV026 to produce pigment while the strains grown in the absence of the extract were able to produce the violacein pigment (Fig. 3).

### *lasR* gene expression in presence or absence of QIFGE.

Analysis of target gene expression relative to an endogenous control gene is a commonly used method to quantify gene expression. According to data analysis, at sub-MIC concentration of QIFGE the expression of gene decreased 8-fold for strain 1 and 4, 4 fold for strain 2 and 5 and 2 fold for strain 3.

## DISCUSSION

*P. aeruginosa* is an opportunistic pathogen mostly associated with nosocomial infections. Serious infections can result in high mortality and morbidity, even with the initiation of the appropriate empiric therapy (1, 2). Like many Gram-negative bacteria, in *P. aeruginosa* production of virulence factors is regulated by QS system. Among AHL mediated Qs systems identified in *P. aeruginosa*, *lasR* which encodes a LuxR-transcriptional protein, is often defined as being the most important in the QS hierarchy (27). Motility by means of flagella causing swarming on solid surface and type IV pili mediated twitching motility are also affected by this system (28).

The results of this study showed that the extract significantly reduced the level of LasA, LasB, swarming motility, twitching motility and biofilm formation (*P*≤0.001). Anti QS-activities using *C. violaceum* CV 026 as a preliminary bioassay were performed in presence or absence of the extract. All the bacterial strains showed reduction in the AHL production by *C. violaceum* CV026 bioassay method in presence of QIFGE in comparison with the bacteria grown in the absence of the extract. Presences of quorum sensing inhibitors have also been reported in several other plant extracts (10, 12, 14, 17). However, in most of these investigations, only one bacterial strain was tested and the effective dose of the extract was much higher and about 10 times more than the QIFGE in this study, Therefore, QIFGE could be a good candidate for further studies in this respect.

In the current study, QIFGE decreased the expression of *lasR* gene in *P. aeruginosa* strains. Studies that investigate effects of plant extracts on gene expression of QS are rare. Similar to our study, Singh and colleagues suggested that *Lagerstroemia speciosa* extract caused down regulation of quorum sensing (QS)-related genes such as *las* and *rhl* (14), which should be similar to this study. In the present study the effect of the extract was not similar for all bacterial strains, and we found a significant variability in strain to strain response in the level of LasB, twitching motility and LasR expression (*P*≤0.001). This is in agreement with Fonseca et al. who reported a considerable strain - to-strain variability in cell surface hydrophobicity, motility and biofilm formation in *P. aeruginosa* in presence of 0.5 MIC of piperacillin/tazobactam (29). It is interesting to note that all the virulence factors tested in strain PAO1, except for LasB, were less affected by QIFGE compared to other strains tested. This can be due to the observation that in strain PAO1, *lasR* is a key regulator in expression of *las*B gene (27). However, for *P. aeruginosa* strain ATCC 27853 in which the activity of *las*B was less affected, the expression of *lasR* showed 8-fold reductions, this could also be due to the presence of other QS signals such as factor-2 as was proposed by Pearson (30).

## CONCLUSION

In this study, QIFGE at sub-MIC concentration decreased the expression of LasA, LasB, swarming, and twitching motility. This extract had inhibitory effect on biofilm formation, AHL production and *lasR* expression significantly, with a relatively low dose and in different strains of *P. aeruginosa*. The strain to strain variability in some cases is important and needs to be evaluated. For further work it is important to fractionate the extract to see the active component and to see if there is a certain fraction capable of this function or whether combinations of different fractions are needed. As a novel plant with a quorum quenching activities on *P. aeruginosa*, one of the leading causes of nosocomial infection, toxicological tests on animal models should be performed before it can be recommended as a therapeutic agent.
